# GPR43 regulation of mitochondrial damage to alleviate inflammatory reaction in sepsis

**DOI:** 10.18632/aging.203572

**Published:** 2021-09-28

**Authors:** Weiwei Zhang, Wusan Wang, Maodi Xu, Haitang Xie, Zhichen Pu

**Affiliations:** 1 Department of Pharmacy, Second Affiliated Hospital of Wannan Medical College, Wuhu 241001, Anhui, China; 2 Department of Pharmacology, College of Pharmacy, Wannan Medical College, Wuhu 241002, Anhui, China; 3 Drug Clinical Evaluation, Yijishan Hospital of Wannan Medical College, Wuhu 241001, Anhui, China; 4 State Key Laboratory of Natural Medicines, Key Lab of Drug Metabolism and Pharmacokinetics, China Pharmaceutical University, Nanjing 210009, Jiangsu, China

**Keywords:** GPR43, ROS, mitochondrial damage, NLRP3 inflammasome, PPARγ

## Abstract

Sepsis is a common critical illness in ICU and always a great difficulty in clinical treatment. GPR43 (G protein-coupled receptor 43) participates in regulating appetite and gastrointestinal peptide secretion to modulate fat decomposition and formation. However, the biological contribution of GPR43 on inflammation of sepsis has not been previously investigated. We investigated the mechanisms of GPR43 gene, which plays a possible role in distinguishing sepsis and contributes to the pathogenesis of sepsis-induced inflammatory reaction. Furthermore, we performed studies with mice induced to sepsis by Cecal Ligation and Puncture (CLP), Knockout GPR43 (GPR43-/-) mice, and Wild Type (WT) mice induced with CLP. In addition, lung tissues and cell samples were analyzed by histology, Quantitative Polymerase Chain Reaction (Q-PCR), Enzyme-linked Immunosorbent (ELISA) Assay, and western blot. GPR43 agonist could significantly reduce inflammation reactions and trigger lung injury in mice with sepsis. As for GPR43-/- mice, the risks of sepsis-induced inflammatory reactions and corresponding lung injury were promoted. On the one hand, the up-regulation of GPR43 gene reduced ROS mitochondrial damage to inhibit inflammatory reactions via the inactivation of NLRP3 Inflammasome by PPARγ/ Nox1/EBP50/ p47phox signal channel. On the other hand, the down-regulation of GPR43 promoted inflammatory reactions *in vitro* model through the acceleration of ROS-dependently mitochondrial damage by PPARγ/ Nox1/EBP50/ p47phox/ NLRP3 signal channel. These findings indicate that the inhibition of GPR43 as a possible important factor of sepsis may shed lights on the mechanism of sepsis-induced inflammation reaction.

## INTRODUCTION

Although the pathogenesis and therapeutic approach of sepsis have been intensively investigated and explored, it is still one of the main causes of death in emergency or intensive care unit (ICU) [1]. Accordingly, there are 20 to 30 million sepsis patients worldwide annually, and the mortality rate is as high as 25% [2]. Existing studies have shown that early diagnosis of sepsis can not only decrease the medical cost of patients, but also play a vital role in improving prognosis [3, 4].

Sepsis-induced acute lung injury (ALI) is a common critical illness in ICU [5]. If the case of rapid disease deterioration and progression into acute respiratory distress syndrome (ARDS), the mortality rate is extremely high, which is always a great difficulty in clinical treatment [6]. A large number of inflammatory cells infiltrate and accumulate in the lung in ALI, along with enhanced expression of various inflammatory mediators and cytokines in the lung [6, 7]. The imbalance of anti-inflammatory and pro-inflammatory balance is a key factor causing lung injury [7].

Infection-caused sepsis can easily progress to multiple organ dysfunction syndrome if not diagnosed and treated in time [8]. Studies have shown that mitochondrial dysfunction plays an important role in the initiation of sepsis course [8–10]. Injured mitochondria can mediate inflammatory responses by promoting the activity of NLRP3 inflammasome [9, 10].

G protein-coupled receptor 43 (GPR43) belongs to the G protein-coupled receptors (GPCR) family [11]. Research on GPR43 has received increasing attention over the past 10 years due to its relationship with fat and glucose metabolism [12]. GPR43 is distributed in different tissues and cells such as adipose tissues, immune cells and gastrointestinal environment [13]. It can be activated by the short-chain fatty acid (SCFA) and is also referred to as FFAR2 according to its endogenous ligand [14]. SCFA is mainly produced by anaerobic bacteria in the small intestine during the fermentation process of starch and indigestible fibers [15]. Research also finds that GPR43 is highly expressed in neutrophils, eosinophils and monocytes, and these immune cells play important roles in the pathophysiological mechanisms of various inflammatory diseases [16]. It has been verified in mice that, the SCFA-induced activation of GPR43 is involved in the chemotaxis of neutrophils, but the mechanism remains unclear. We investigated the mechanisms of GPR43 gene, a possible role for distinguish sepsis, contributes to the pathogenesis of sepsis-induced inflammatory reaction.

## RESULTS

### The inhibition of GPR43 gene was associated with future risk of sepsis-induced inflammatory reactions

**Figure 1:**
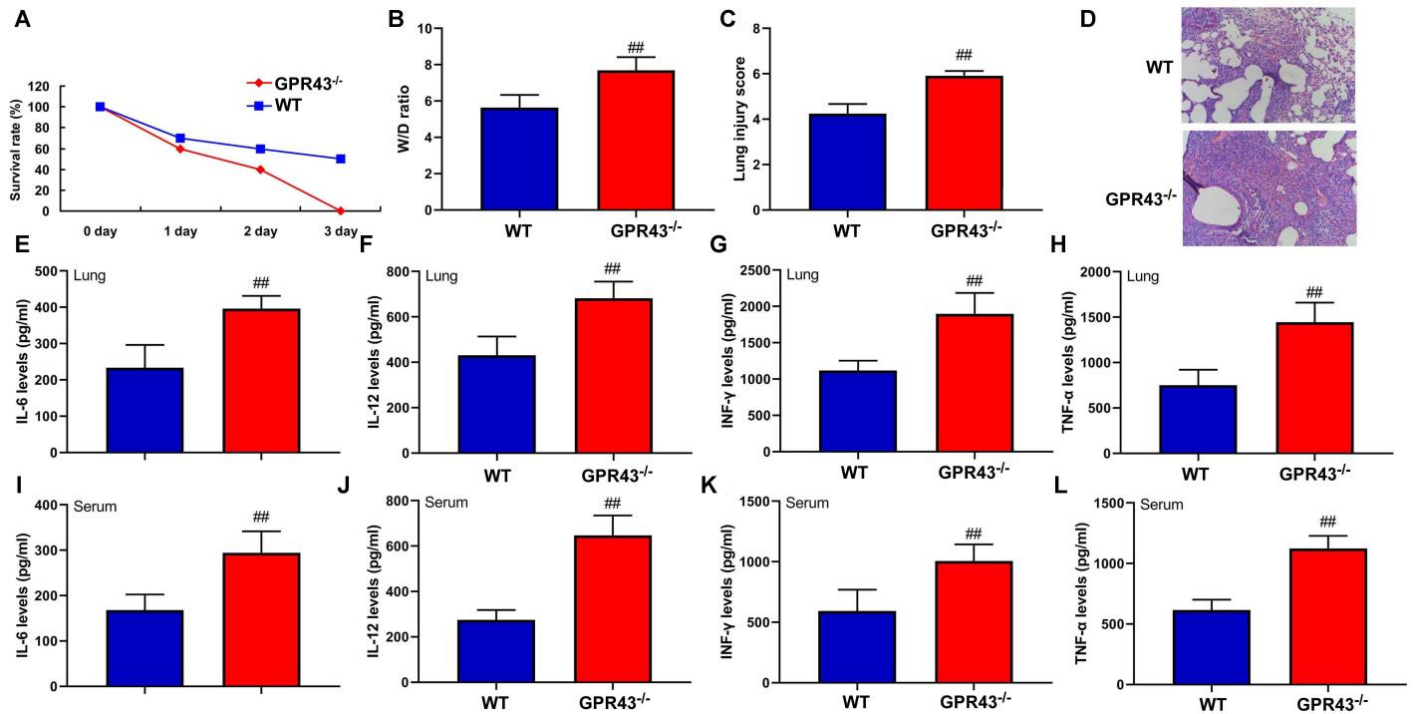
**The inhibition of GPR43 gene is associated with future risk of sepsis-induced inflammatory reactions.** Survival rate (**A**) in tissue of CLP mice for 72 h; W/D rate (**B**), lung injury score (**C**) and lung tissue using HE staining (**D**) in CLP mice for 24 h; IL-6 (**E**), IL-10 (**F**), IL-12 (**G**) and INF-γ (**H**) levels in tissue of CLP mice for 24 h; IL-6 (**I**), IL-10 (**J**), IL-12 (**K**) and INF-γ (**L**) levels in serum of CLP mice for 24 h. WT, WT mice with CLP; GPR43^-/-^, GPR43^-/-^ mice with CLP. ##p<0.01 compared with WT mice with CLP.

Firstly, in order to better understand the function of GPR43 gene in sepsis-induced inflammatory reactions, GPR43^-/-^ mice were induced by CLP. The inhibition of W/D rate and lung injury score, the recovery of survival rate, and the reduction of IL-6, IL-10, IL-12 and INF-γ levels in tissue and serum were more effectively observed in GPR43^-/-^ mice with CLP, in comparison to WT mice with CLP (Figure 1). According to the results, GPR43 gene inhibition is a pathogenic factor of sepsis which can activate inflammatory responses in the process of sepsis model.

**Figure 2:**
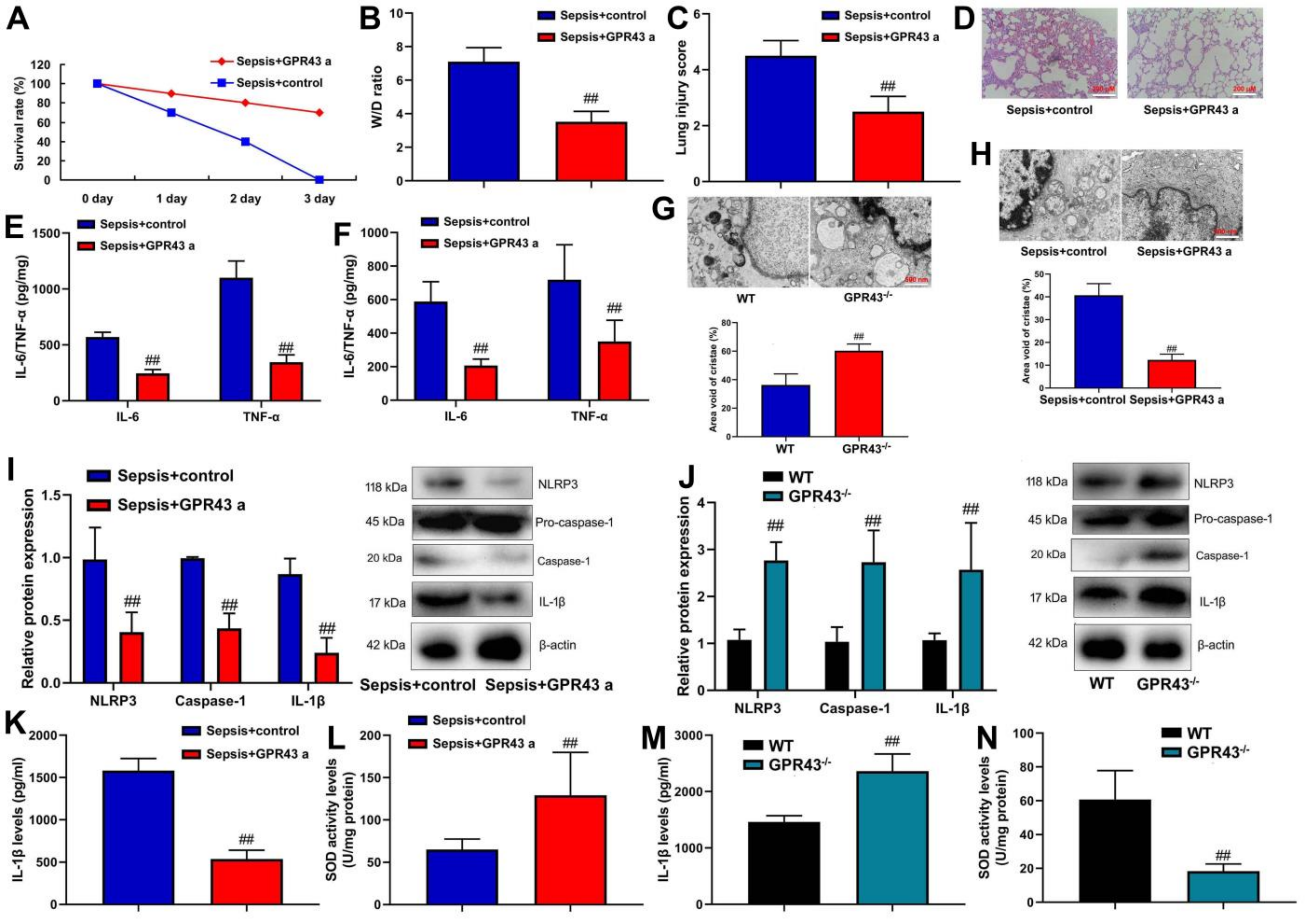
**GPR43 agonist is involved in sepsis-induced inflammatory reactions through trigger NLRP3 inflammasome.** Survival rate (**A**) in CLP mice with GPR43 agonist (4-CMTB, 10 mg/kg, i.p.) for 72 h; W/D rate (**B**), lung injury score (**C**) and lung tissue using HE staining (**D**) in CLP mice with GPR43 agonist (4-CMTB, 10 mg/kg, i.p.) for 24 h; IL-6/IL-10 levels in tissue of CLP mice (**E**) in CLP mice with GPR43 agonist (4-CMTB, 10 mg/kg, i.p.) for 24 h; IL-6/IL-10 levels in serum of CLP mice (**F**) in CLP mice with GPR43 agonist (4-CMTB, 10 mg/kg, i.p.) for 24 h; Representative electron microscopy images and area void of cristae (minimum of 40 mitochondria) was used to measure mitochondrial cristae density in macrophage CLP of mice with GPR43 agonist (4-CMTB, 10 mg/kg, i.p.) (**G**) for 24 h; Representative electron microscopy images and area void of cristae (minimum of 40 mitochondria) was used to measure mitochondrial cristae density in macrophage CLP of mice with GPR43 agonist (4-CMTB, 10 mg/kg, i.p.) (**H**) for 24 h; NLRP3, Caspase-1 and IL-1β protein expressions in CLP mice with GPR43 agonist (4-CMTB, 10 mg/kg, i.p.) (**I**) for 24 h; NLRP3, Caspase-1 and IL-1β protein expressions in GPR43^-/-^ mice of CLP (**J**) for 24 h; Serum IL-1β and SOD levels in CLP mice with GPR43 agonist (4-CMTB, 10 mg/kg, i.p.) (**K**, **L**) for 24 h; Serum IL-1β and SOD levels in GPR43^-/-^ mice of CLP (**M**, **N**) for 24 h. Sepsis+control, CLP mice with normal saline; Sepsis+GRP43 a, CLP mice with i GPR43 agonist (4-CMTB, 10 mg/kg, i.p.); WT, WT mice with CLP; GPR43^-/-^, GPR43^-/-^ mice with CLP. ##p<0.01 compared with WT mice with CLP or ##p<0.01 compared with CLP mice with normal saline.

GPR43 agonist presented abdominal macrophage to induce NLRP3 in the model of sepsis. In order to investigate the small molecular substances in the body of GPR43 gene involved in sepsis-induced inflammatory responses, GPR43 agonist (4-CMTB, 10 mg/kg, i.p.) was used to improve the model of sepsis-induced inflammatory reactions. It could be found that GPR43 agonist significantly restored W/D rate and lung injury score, decreased survival rate, and repressed tissue and serum of IL-6/IL-10 levels in CLP mice (Figure 2A–2F). Meanwhile, GPR43^-/-^ significantly aggravated mitochondrial damage and triggered mitochondrial fission (including MFN2 and MIGA2 protein) in abdominal macrophage of CLP mice (Figure 2G and Supplementary Figure 1A). However, GPR43 agonist reversed the function of inflammatory reactions in abdominal macrophage of sepsis model (Figure 2H and Supplementary Figure 1B). Besides, we validated that the increased expression of GPR43 decreased the risk inflammatory reactions of sepsis through regulation of mitochondrial fission, and its function was unclear.

Indeed, NLRP3 Inflammasome is known for regulation of inflammation in sepsis model, but whether GPR43 regulates NLRP3 Inflammasome remains unclear. GPR43 agonist observably suppressed NLRP3, caspase-1 and IL-1β protein expressions in CLP mice (Figure 2I). At the same time, there was an increases of NLRP3, caspase-1 and IL-1β protein expressions in GPR43^-/-^ mice of CLP model (Figure 2J). More specifically, GPR43 agonist significantly decreased serum IL-1β levels, and promoted SOD activity levels in CLP mice (Figure 2K, 2L). The activation of IL-1β level and the inhibition of SOD activity levels were conspicuously observed in GPR43^-/-^ mice of CLP model (Figure 2M, 2N). However, our results excluded a point that the inhibition of GPR43 promoted inflammatory reactions in sepsis model by the activation of NLRP3 inflammasome, and its activation mechanism was unclear.

### GPR43 gene triggered NLRP3 inflammasome in macrophage by regulation of mitochondrial fission

**Figure 3:**
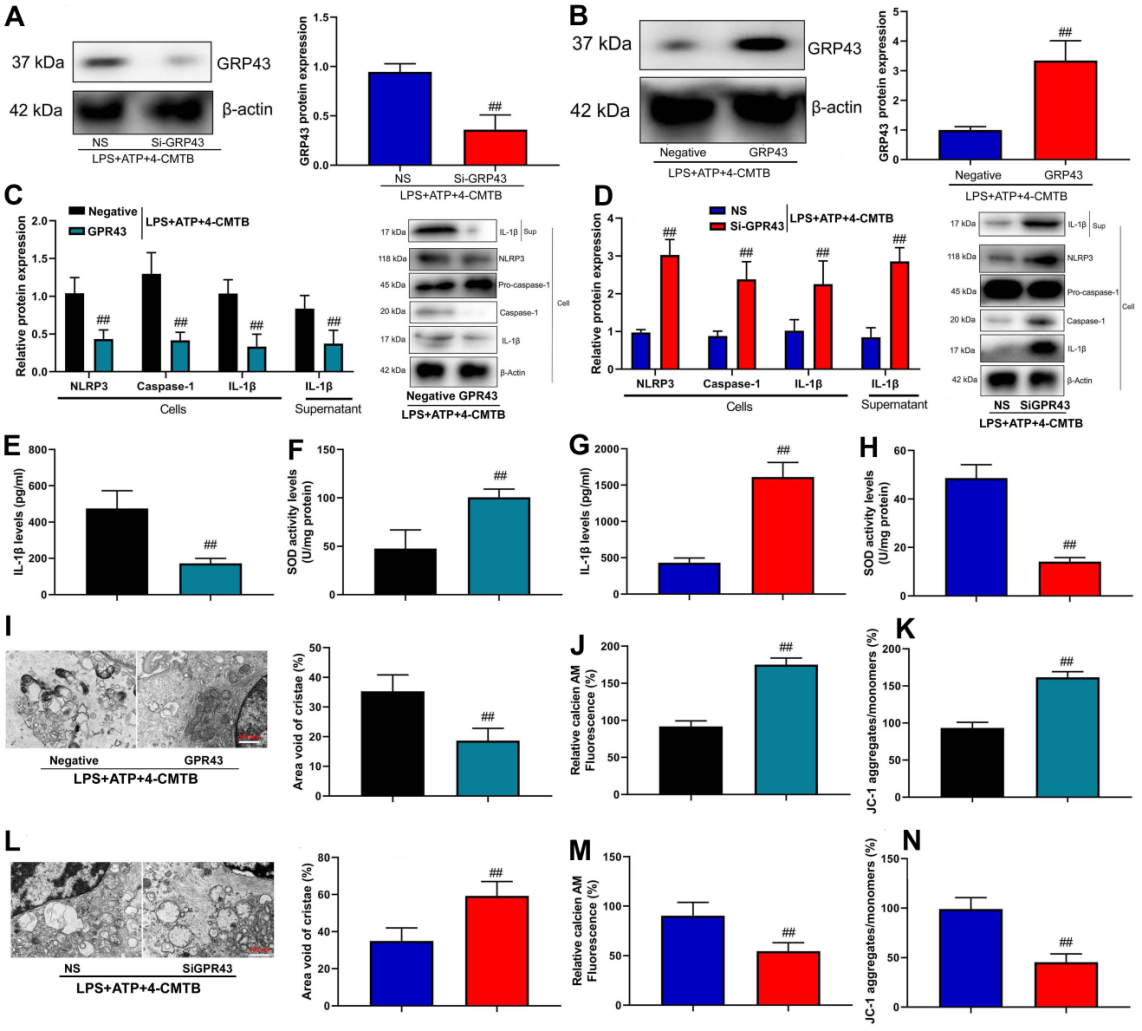
**GPR43 gene trigger NLRP3 inflammasome in macrophage by regulation of mitochondrial fission.** GPR43 protein expression in macrophage by down-regulation of GPR43 and LPS+ATP+GPR43 agonist (**A**); GPR43 protein expression in macrophage by up-regulation of GPR43 and LPS+ATP+GPR43 agonist (**B**); NLRP3, Caspase-1 and IL-1β protein expressions in cells and IL-1β protein expression in macrophage by up-regulation of GPR43 and LPS+ATP+GPR43 agonist (**C**); NLRP3, Caspase-1 and IL-1β protein expressions in cells and IL-1β protein expression in macrophage by down-regulation of GPR43 and LPS+ATP+GPR43 agonist (**D**); IL-1β and SOD levels in macrophage by up-regulation of GPR43 and LPS+ATP+GPR43 agonist (**E**, **F**); IL-1β and SOD levels in macrophage by down-regulation of GPR43 and LPS+ATP+GPR43 agonist (**G**, **H**); Representative electron microscopy images, area void of cristae (minimum of 40 mitochondria) was used to measure mitochondrial cristae density (**I**), Calcein-AM/CoCl2 assay (**J**), and Calcein-AM/CoCl2 assay and dissipation of Δψm by JC-1 assay (**K**) in macrophage by up-regulation of GPR43 and LPS+ATP+GPR43 agonist; Representative electron microscopy images, area void of cristae (minimum of 40 mitochondria) was used to measure mitochondrial cristae density (**L**), Calcein-AM/CoCl2 assay (**J**), and Calcein-AM/CoCl2 assay and dissipation of Δψm by JC-1 assay (**M**) in macrophage by down-regulation of GPR43 and LPS+ATP+GPR43 agonist (**N**). Negative, negative control; GPR43, over-expression of GPR43; NS, si-negative control; Si-GPR43, down-regulation of GPR43; LPS+ATP+4-CMTB, macrophage by treated with LP+ATP+4-CMTB. ##p<0.01 compared with negative control or si-negative control.

We investigated the mechanism of how GPR43 gene triggered NLRP3 Inflammasome in sepsis-induced inflammatory reactions. Macrophage was an important proinflammatory cytokine of sepsis [17]. In addition, GPR43 plasmid induced GPR43 mRNA expression in macrophage, and si-GPR43 suppressed GPR43 mRNA expression in macrophage (Supplementary Figure 1C, 1D). Meanwhile, Si-GPR43 suppressed GPR43 protein expression in macrophage by LPS+ATP+GPR43 agonist (4-CMTB, 20 μM) (Figure 3A), and the GPR43 plasmid induced GPR43 protein expression in macrophage by GPR43 agonist (Figure 3B). Over-expression of GPR43 not only suppressed the cell expressions of NLRP3, caspase-1 and IL-1β protein, but also decreased supernatant of IL-1β protein expression in macrophage by LPS+ATP+GPR43 agonist (Figure 3C). Apart from that, the inhibition of GPR43 induced NLRP3, caspase-1 and IL-1β cell protein expressions, and activated supernatant of IL-1β protein expression in macrophage by LPS+ATP+GPR43 agonist (Figure 3D). Interestingly, over-expression of GPR43 reduced supernatant IL-1β levels, and increased SOD activity levels in macrophage by LPS+ATP+GPR43 agonist (Figure 3E, 3F). Unexpectedly, the down-regulation of GPR43 induced supernatant IL-1β levels, and reduced SOD activity levels in macrophage by LPS+ATP+GPR43 agonist (Figure 3G, 3H). Moreover, over-expression of GPR43 restored mitochondrial damage and mitochondrial fission in macrophage by LPS+ATP+GPR43 agonist (Figure 3I–3K and Supplementary Figure 1E). More specifically, the down-regulation of GPR43 aggravated mitochondrial damage and mitochondrial fission in macrophage by LPS+ATP+GPR43 agonist (Figure 3L–3N and Supplementary Figure 1F).

As shown by our results, NLRP3 inflammasome activity was triggered by GPR43 gene in sepsis-induced inflammatory reactions model. Next, we explored whether NLRP3 Inflammasome is an important target of GPR43 in sepsis-induced inflammatory reactions model. As expected, NLRP3 inhibitor (20 mg/ kg of INF39) improved survival rate, but diminished W/D rate and lung injury score in GPR43^-/-^ mice with CLP (Supplementary Figure 2A–2D). Additionally, NLRP3 inhibitor decreased IL-1β levels and suppressed NLRP3, caspase-1 and IL-1β protein expressions in GPR43^-/-^ mice with CLP (Supplementary Figure 2E, 2F). More specifically, si-NLRP3 suppressed NLRP3, caspase-1 and IL-1β protein expression *in vitro* model (Supplementary Figure 3A). In contrast, si-NLRP3 reduced supernatant IL-1β levels, and suppressed NLRP3, caspase-1 and IL-1β protein expressions in macrophage by LPS+ATP+GPR43 agonist (Supplementary Figure 2G). These results indicated that GPR43 exerted a direct regulatory effect on NLRP3 activation in sepsis-induced inflammatory reactions by regulating mitochondrial fission.

### The inhibition of GPR43 activated NLRP3 inflammasome by ROS production-induced mitochondrial fission

**Figure 4:**
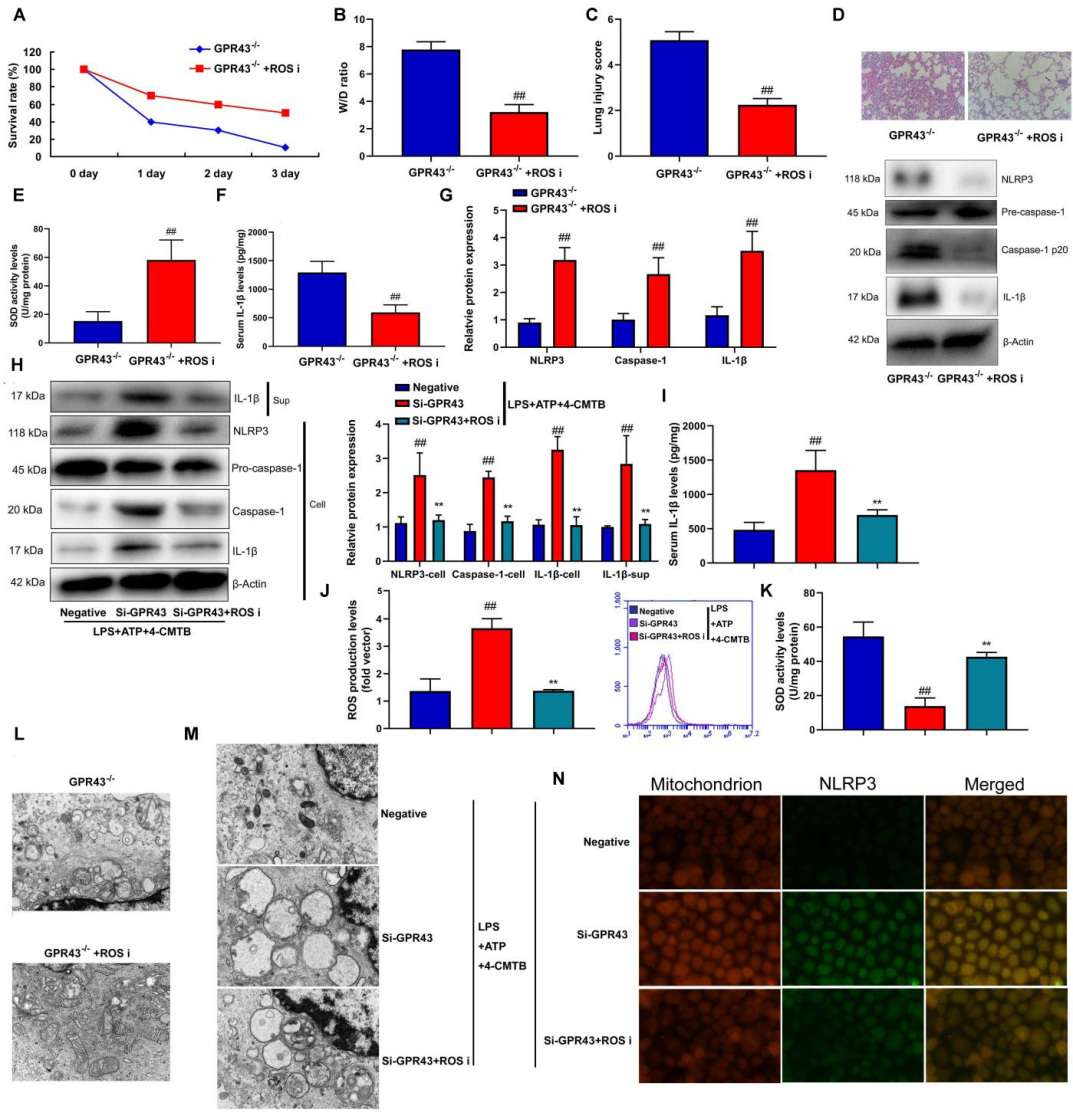
**The inhibition of GPR43 activate NLRP3 inflammasome by ROS production-induced mitochondrial fission.** Survival rate (**A**) in GRP43^-/-^ mice with CLP and ROS I for 72 h; W/D rate (**B**), lung injury score (**C**), lung tissue using HE staining (**D**), SOD activity level (**E**), serum IL-1β levels (**F**), NLRP3/caspase-1/ IL-1β protein expressions (**G**) in GRP43^-/-^ mice with CLP and ROS I for 24 h; NLRP3, Caspase-1 and IL-1β protein expressions in cells and IL-1β protein expression in supernatant (**H**), IL-1β levels (**I**), ROS production level (**J**), and SOD activity levels (**K**) in macrophage by down-regulation of GPR43 and LPS+ATP+GPR43 agonist for 24 h; Representative electron microscopy images (**L**) in macrophage of GRP43^-/-^ mice with CLP for 24 h; Representative electron microscopy images (**M**) in macrophage by down-regulation of GPR43 and LPS+ATP+GPR43 agonist for 24 h; Confocal showed the accumulation of ROS production within mitochondria (**N**) in macrophage by down-regulation of GPR43 and LPS+ATP+GPR43 agonist for 24 h. GPR43^-/-^, GPR43^-/-^ mice with CLP; GPR43^-/-^+ROS i, GPR43^-/-^ mice of CLP with ROS inhibitor; Negative, negative control; Si-GPR43, down-regulation of GPR43; LPS+ATP+4-CMTB, macrophage by treated with LPS+ATP+4-CMTB. ##p<0.01 compared with GPR43^-/-^ mice with CLP or GPR43^-/-^ mice with CLP; **p<0.01 compared with down-regulation of GPR43.

**Figure 5:**
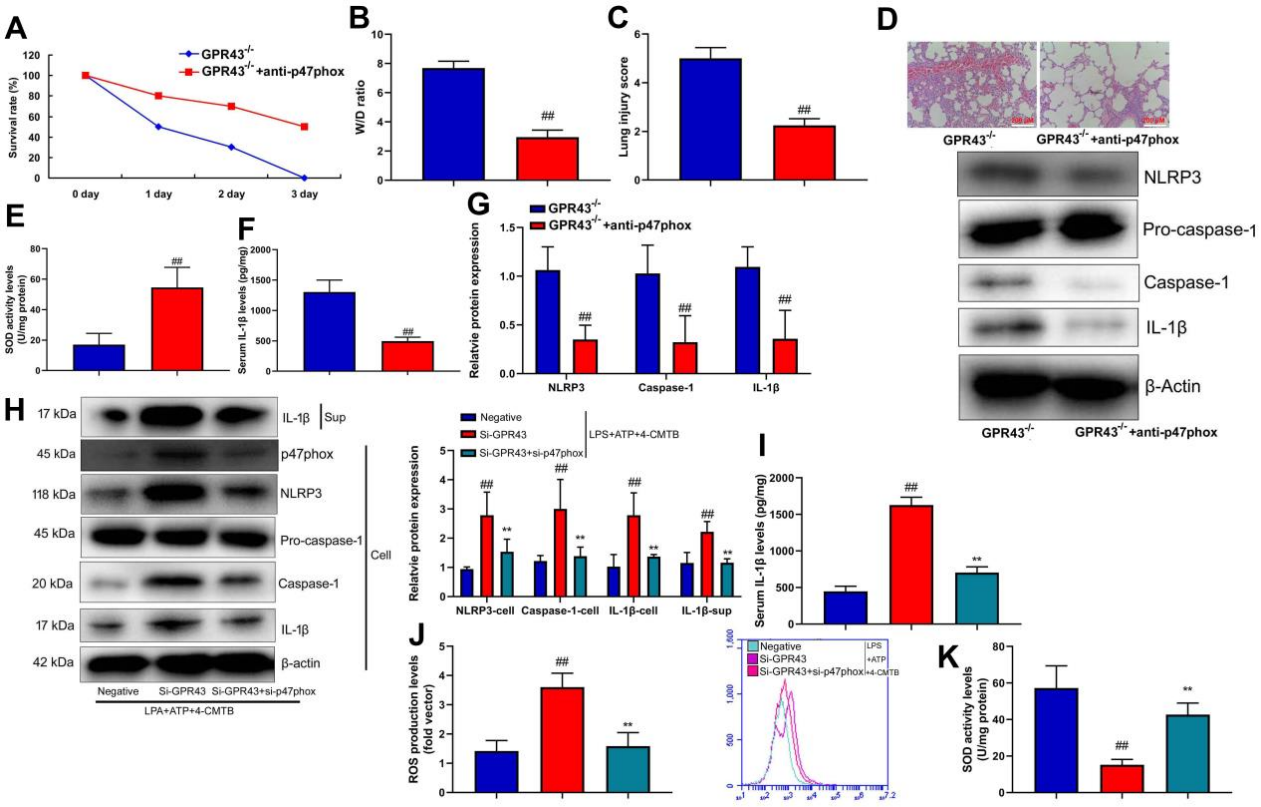
**P47phox caused ROS production in the function of GPR43 in sepsis-induced inflammatory reactions model.** Survival rate (**A**) in GRP43^-/-^ mice with CLP and anti-p47phox for 72 h; W/D rate (**B**), lung injury score (**C**), lung tissue using HE staining (**D**), SOD activity level (**E**), serum IL-1β levels (**F**), NLRP3/caspase-1/ IL-1β protein expressions (**G**) in GRP43^-/-^ mice with CLP and anti-p47phox for 24 h; NLRP3, Caspase-1 and IL-1β protein expressions in cells and IL-1β protein expression in supernatant (**H**), IL-1β levels (**I**), ROS production level (**J**), and SOD activity levels (**K**) in macrophage by down-regulation of GPR43 and LPS+ATP+GPR43 agonist for 24 h. GPR43^-/-^, GPR43^-/-^ mice with CLP; GPR43^-/-^+ROS i, GPR43^-/-^ mice of CLP with ROS inhibitor; Negative, negative control; Si-GPR43, down-regulation of GPR43; si-p47phox, down-regulation of p47phox; LPS+ATP+4-CMTB, macrophage by treated with LPS+ATP+4-CMTB. ##p<0.01 compared with GPR43^-/-^ mice with CLP or GPR43^-/-^ mice with CLP; **p<0.01 compared with down-regulation of GPR43.

In order to determine the potential mechanisms by which the inhibition of GPR43 activated the NLRP3 inflammasome, ROS production was defined as the key mediators in activation of the NLRP3 inflammasome [18]. It could be found that the levels of ROS production in abdominal macrophage of GPR43^-/-^ mice of sepsis were more markedly observed, in comparison to WT mice of sepsis group (Supplementary Figure 3B). That is to say, ROS might influence the activation of the NLRP3 inflammasome by GPR43 expression. In order to verify whether ROS production is necessary for activation of NLRP3 Inflammasome activity by the inhibition of GPR43 expression, GPR43^-/-^ mice with CLP were treated with ROS inhibitor (100 mg/kg of N-Acetylcysteine amide, i.p.). As a result, ROS inhibitor raised survival rate, and lowered W/D rate and lung injury score in GPR43^-/-^ mice with CLP (Figure 4A–4D). By contrast, ROS inhibitor augmented SOD activity level, reduced IL-1β levels, and suppressed NLRP3, caspase-1 and IL-1β protein expressions in GPR43^-/-^ mice with CLP (Figure 4E–4G). As expected, ROS inhibitor (500 μM of N-Acetylcysteine amide) not only weakened supernatant IL-1β levels, but also abated NLRP3, caspase-1 and IL-1β protein expressions *in vitro* model (Supplementary Figure 3C). For one thing, ROS inhibitor weakened supernatant IL-1β levels and abated NLRP3, caspase-1 and IL-1β protein expressions in macrophage by the down-regulation of GPR43 and LPS+ATP+GPR43 agonist (Figure 4H, 4I). For another, ROS inhibitor elevated SOD activity level and reduced ROS production level in macrophage by down-regulation of GPR43 and LPS+ATP+GPR43 agonist (Figure 5J, 5K). ROS inhibitor not only greatly reduced mitochondrial damage in abdominal macrophage of GPR43^-/-^ mice with CLP (Figure 4L), but also memorably inhibited mitochondrial damage in macrophage by down-regulation of GPR43 and LPS+ATP+GPR43 agonist (Figure 4M). According to the result of confocal analysis, ROS inhibitor reduced the accumulation of NLRP3 protein within mitochondria in macrophage by down-regulation of GPR43 and LPS+ATP+GPR43 agonist (Figure 4N). In short, mitochondrial damage caused by ROS production represented a general mechanism that GPR43 induced NLRP3 Inflammasome activity in sepsis-induced inflammatory reactions model.

### P47phox caused ROS production in the function of GPR43 in sepsis-induced inflammatory reactions model

Furthermore, we analyzed whether p47phox was functionally involved in ROS production and triggered NLRP3 inflammasome activity in the function of GPR43 on sepsis-induced inflammatory reactions. Anti-p47phox body (100 ng/mice) increased survival rate, but reduced W/D rate and lung injury score in GPR43^-/-^ mice with CLP (Figure 5A–5D). Moreover, SOD activity level was increased, and NLRP3 Inflammasome protein complex assembly was blocked by anti-p47phox body in GPR43^-/-^ mice with CLP (Figure 5E–5G). Next, si-p47phox reduced the activation of supernatant IL-1β levels as well as the induction of NLRP3, caspase-1 and IL-1β protein expressions *in vitro* model (Supplementary Figure 3D). The study further determined whether p47phox triggered ROS-induced mitochondrial damage in the function of GPR43 in sepsis-induced inflammatory reactions model. Beyond that, Si-p47phox increased SOD activity levels, but reduced ROS production in macrophage by down-regulation of GPR43 and LPS+ATP+GPR43 agonist (Figure 5J, 5K). Therefore, p47phox activated ROS-induced mitochondrial damage to cause GPR43 inhibition to induce NLRP3 Inflammasome activity in sepsis-induced inflammatory reactions model.

### Nox1/EBP50/p47phox was involved in the activation of NLRP3 inflammasome by GRP43 gene in sepsis model

**Figure 6:**
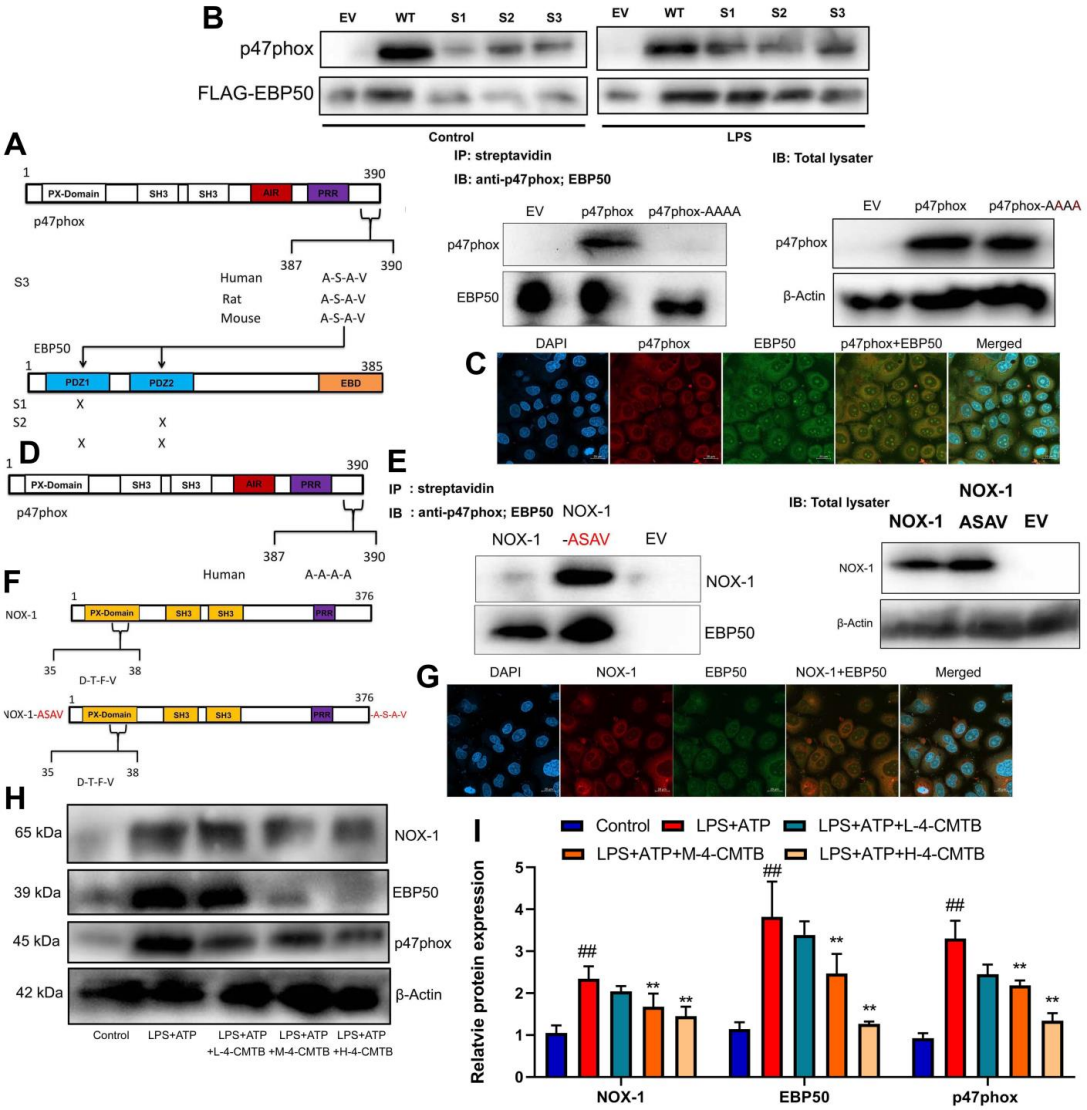
**Nox1/EBP50/p47phox is involved in the activation of NLRP3 inflammasome by GRP43 gene in sepsis model.** EBP50 and p47phox sequence structures highlighting the PDZ domains of EBP50 and the potential PDZ-binding motif on p47phox, streptavidin regulated anti-p47phox or anti-EBP50 antibodies on RAW264.7 cell (**A**); Anti-FLAG antibody with anti-p47phox or anti-FLAG antibodies on lysates (**B**); P47phox and EBP50 expression *in vitro* model using confocal (**C**); Outline of p47phox sequence structures highlighting the mutation (in red) of C-terminal PDZ binding motif on p47phox (**D**); NOX-1 and p47phox sequence structures highlighting the PDZ domains of NOX-1 and the potential PDZ-binding motif on p47phox (**E**); Anti-FLAG antibody with anti- NOX-1 or anti-FLAG antibodies on lysates (**F**); NOX-1 and EBP50 expression *in vitro* model using confocal (**G**); Nox1, EBP50 and p47phox protein expressions in macrophage by down-regulation of GPR43 and LPS+ATP+GPR43 agonist (**H**, **I**). Control, control group; LPS+ATP, macrophage by treated with LPS+ATP; L-4-CMTB, 10 μM of 4-CMTB; M-4-CMTB, 20 μM of 4-CMTB; H-4-CMTB, 40 μM of 4-CMTB. ##p<0.01 compared with control group; **p<0.01 compared with LPS+ATP.

In this study, mutant EBP50 plasmids lacking active PDZ1, PDZ2, or both were used to check the interaction between EBP50 and p47phox involving PDZ domains (Figure 6A). It was found that the docking of the C-terminal 4 leftovers motif of p47phox within the ligand mechanism of PDZ1 of EBP50 was advantageous (Figure 6B). Confocal analysis indicated that p47phox protein was bound with EBP50 protein expression *in vitro* model (Figure 6C).

NoxO1 in its native form lacked the A-S-A-V motif. In this study, a NoxO1 mutant was supplemented into C-terminal A-S-A-V motif so as to examine whether this motif was sufficient for EBP50 interaction (Figure 6D). As shown by these results, NoxO1 was a weak association with EBP50, which might be attributed to a rare and unusual potential PDZ binding motif deep into its sequence and close to the N-terminal (Figure 6E). At the same time, the association with EBP50 was significantly enhanced in the cells expressing NoxO1 ASAV (Figure 6F). Confocal analysis revealed that NoxO1 protein was bound with EBP50 protein expression *in vitro* model (Figure 6G). Thus, these data supported that the A-S-A-V motif at the c-terminal of p47phox was the key and sufficient motif for EBP50 binding. Then, it was found that 4-CMTB (10, 20 and 40 μM) suppressed Nox1, EBP50 and p47phox protein expressions in macrophage by LPS+ATP (Figure 6H, 6I).

This study identified a possible signal pathway that activated NLRP3 Inflammasome to secrete IL-1β levels under sepsis conditions. We further analyzed whether such the function of GRP43 triggered by NOX-1 was pathophysiologically relevant. It could be found that GRP43^-/-^ mice with CLP and NOX-1 inhibitor (0.05 μM of ML171) were characterized by significant up-regulation of survival rate as well as inhibition of W/D rate and lung injury score (Supplementary Figure 4A–4D). The inhibition of serum IL-1β levels together with the suppression of p47phox, EBP50, NLRP3, caspase-1 and IL-1β protein expressions were observed at GRP43^-/-^ mice with CLP and NOX-1 inhibitor (Supplementary Figure 4E–4G). Notably, si-NOX-1 was adopted to reduce the expression of NOX-1 protein *in vitro* model (Supplementary Figure 3E). Through down-regulation of GPR43 and LPS+ATP+GPR43 agonist, Si-NOX-1 suppressed the activation supernatant of IL-1β levels, upstream p47phox, EBP50, NLRP3, caspase-1 and IL-1β protein in macrophage (Supplementary Figure 4H, 4I). Consistently, Si-NOX-1 also reduced SOD activity level, and decreased ROS production levels in macrophage by down-regulation of GPR43 and LPS+ATP+GPR43 agonist (Supplementary Figure 4J–4L). As revealed by these results, the induction of NLRP3 inflammasome through GPR43 inhibition-caused mitochondrial damage by NOX-1 was dominant in determining sepsis-induced inflammatory reactions.

### GPR43 was involved in the activation of NLRP3 inflammasome in sepsis model by ROS-induced mitochondrial damage via PPARγ

**Figure 7:**
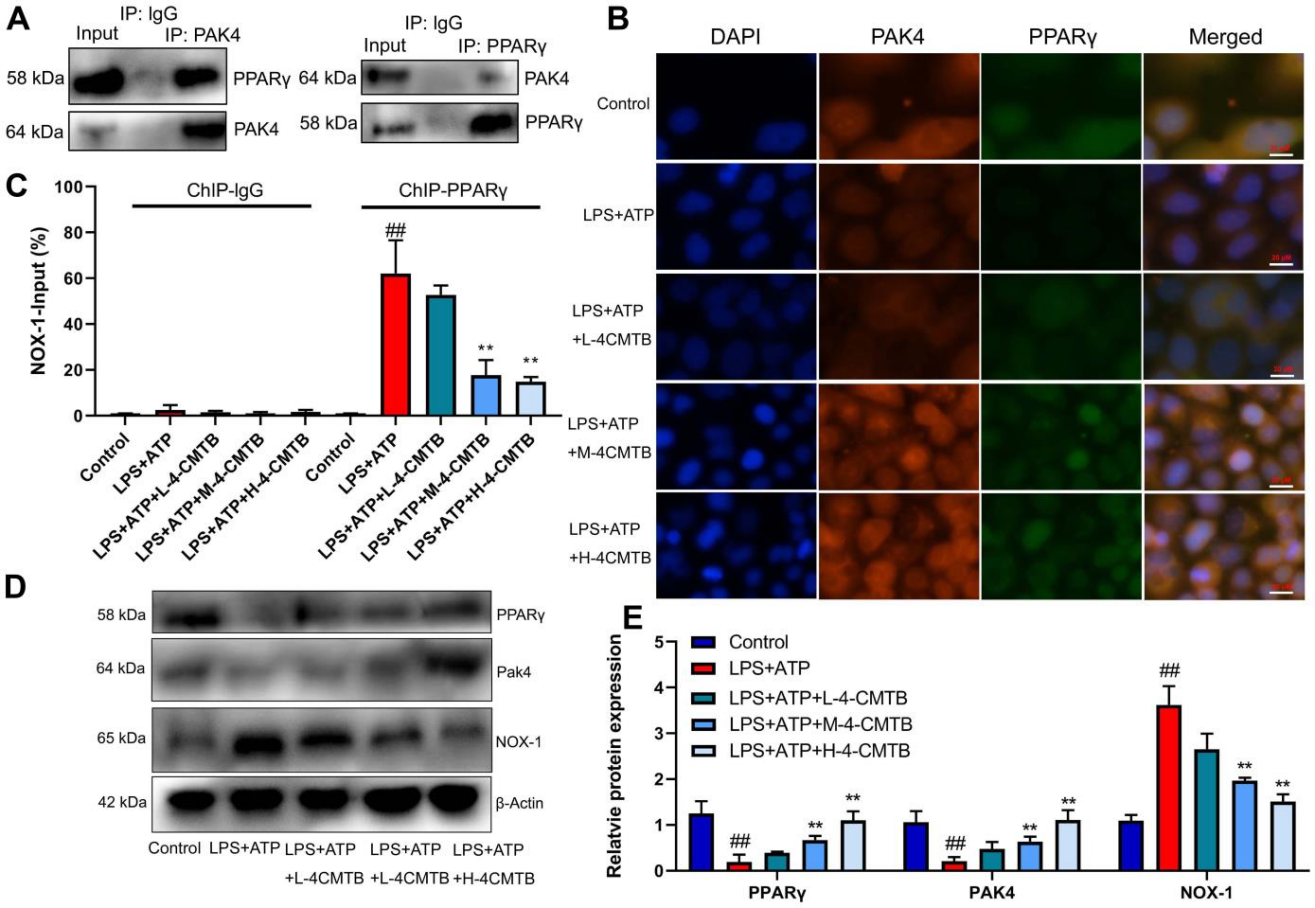
**Interaction of PAK4 with PPARγ to regulate Nox1 expression.** Robust interaction between PAK4 and PPARγ was confirmed by IP (**A**); PAK4 and PPARγ expressions in macrophage by LPS+ATP (**B**); PPARγ binding with the Nox1 promoter in macrophage by LPS+ATP (**C**); PAK4, PPARγ and Nox1 protein expressions in macrophage by LPS+ATP+4-CMTB (**D**, **E**). Control, control group; LPS+ATP+4-CMTB, macrophage by treated with LPS+ATP+4-CMTB. ##p<0.01 compared with control group; **p<0.01 compared with down-regulation of GPR43.

This study validated the mechanism of how GPR43 regulated NLRP3 Inflammasome in sepsis model. The endogenous proteins, which showed robust interaction between PAK4 and PPARγ, were confirmed by IP (Figure 7A). According to confocal analysis, 4-CMTB (20 and 40 μM) not only induced PAK4 and PPARγ expressions, but also promoted PAK4 protein to enter nucleus in macrophage by LPS+ATP (Figure 7B). ChIP assays revealed an increased PPARγ binding with the Nox1 promoter in macrophage by LPS+ATP (Figure 7C). Apart from that, 4-CMTB (20 and 40 μM) not only reduced the PPARγ binding with the Nox1 in macrophage by LPS+ATP (Figure 7C), but also induced PAK4 and PPARγ protein expressions and suppressed Nox1 protein expression in macrophage by LPS+ATP (Figure 7D, 7E). In short, these results demonstrated the association of nuclear-PAK4 with PPARγ which bound Nox1 expression in macrophage by LPS+ATP.

**Figure 8:**
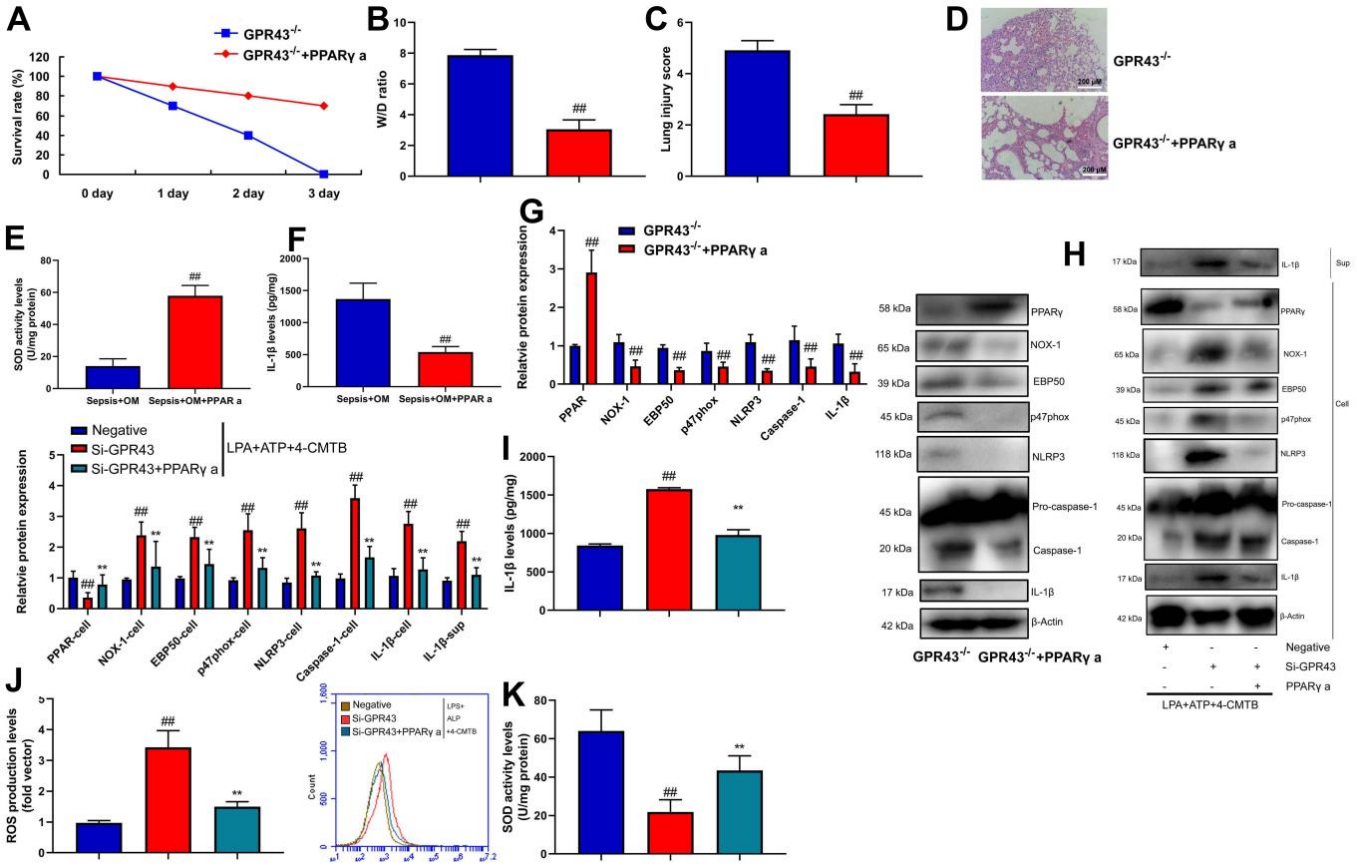
**GPR43 is involved in the activation of NLRP3 inflammasome in sepsis model by PPARγ.** Survival rate (**A**) in GRP43^-/-^ mice with CLP and PPARγ a for 72 h; W/D rate (**B**), lung injury score (**C**), lung tissue using HE staining (**D**), serum IL-1β levels (**E**), PPARγ/NOX-1/EBP50/ p47phox/NLRP3/caspase-1/ IL-1β protein expressions (**F**) in GRP43^-/-^ mice with CLP and PPARγ a for 24 h; PPARγ, NOX-1, EBP50, p47phox, NLRP3, Caspase-1 and IL-1β protein expressions in cells and IL-1β protein expression in supernatant (**G**, **I**), IL-1β levels (**H**), ROS production level (**J**), and SOD activity levels (**K**) in macrophage by down-regulation of GPR43 and LPS+ATP+GPR43 agonist for 24 h. GPR43^-/-^, GPR43^-/-^ mice with CLP; GPR43^-/-^+ PPARγ a, GPR43^-/-^ mice of CLP with PPARγ a; Negative, negative control; Si-GPR43, down-regulation of GPR43; PPARγ a, Pioglitazone; LPS+ATP+4-CMTB, macrophage by treated with LPS+ATP+4-CMTB. ##p<0.01 compared with GPR43^-/-^ mice with CLP or GPR43^-/-^ mice with CLP; **p<0.01 compared with down-regulation of GPR43.

Next, we explored whether PPARγ was functionally involved in GPR43’s induction of NLRP3 Inflammasome in sepsis model. PPARγ agonist (20 mg/kg of Pioglitazone) markedly recovered survival rate, W/D rate, and lung injury score in GRP43^-/-^ mice with CLP (Figure 8A–8D), whereas it markedly reduced serum IL-1β levels, induced PPARγ and Park4 protein expression, and suppressed NOX-1, p47phox, EBP50, NLRP3, caspase-1 and IL-1β protein expressions in GRP43-/- mice with CLP (Figure 8E, 8F). Beyond that, PPARγ agonist (100 nM of Pioglitazone) not only suppressed NOX-1, p47phox, EBP50, NLRP3, caspase-1 and IL-1β protein expressions, but also induced PPARγ and Park4 protein expression *in vitro* model (Supplementary Figure 3F). Meanwhile, PPARγ agonist slightly suppressed NOX-1, p47phox, EBP50, NLRP3, caspase-1 and IL-1β protein expressions, and induced PPARγ and Park4 protein expressions in macrophage by down-regulation of GPR43 and LPS+ATP+GPR43 agonist (Figure 8G–8I), while it slightly reduced SOD activity level, and decreased ROS production levels in macrophage by down-regulation of GPR43 and LPS+ATP+GPR43 agonist (Figure 8J, 8K). To conclude, GPR43 is involved in the inactivation of NLRP3 inflammasome in sepsis model by ROS-induced mitochondrial damage via PPARγ/ EBP50/Nox1/p47phox.

## DISCUSSION

Lungs are generally the most vulnerable organ to be involved in sepsis and can easily cause ALI [19]. The subsequent systemic inflammation is the key to the pathogenesis of ALI. Studies have shown that the pro-inflammatory cytokines (including TNFα, IL6, and macrophage inflammatory protein 2) secreted by inflammatory cells in sepsis-induced ALI can activate neutrophils and vascular endothelial cells to initiate and maintain pulmonary inflammation [17, 20]. GPR43 also participate in regulating the blood lipid concentration and inflammation occurrence process in human body, which is even closely related to cell carcinogenesis [21]. As an important regulatory receptor of glucose and fat metabolism, GPR43 has become a vital drug screening target [22–24]. We observed that the inhibition of W/D rate and lung injury score, the recovery of survival rate and the lower of IL-6, IL-10, IL-12 and INF-γ levels in tissue and serum were effectively observed in GPR43^-/-^ mice with CLP, compared with WT mice with CLP. Meanwhile, GPR43 agonist significantly restored W/D rate and lung injury score, decreased survival rate, and repressed tissue and serum of IL-6/IL-10 levels in CLP mice. Carr et al. elucidated that GPR43 receptor expression is associated with increased in patients with sepsis at 30-day survival [25]. Therefore, these results indicated GPR43 might therefore be developed for treatment of sepsis-induced lung injury. We thought the anti-inflammatory effects of GPR43 is at the middle and late stage or the whole process of inflammation in sepsis. However, the specific anti-inflammatory effects need to be further study.

GPR43 activation by SCFAs can directly inhibit the inflammation of the hypothalamus and suppress appetite, further altering the balance of energy metabolism [26, 27]. Our study establishes that GPR43 gene trigger NLRP3 Inflammasome in macrophage by regulation of mitochondrial fission. Xu et al. demonstrated that acetate regulates the NLRP3 inflammasome via GPR43 [28]. Our results support that GPR43 is involved in the activation of NLRP3 inflammasome in macrophage of sepsis through the regulation of mitochondrial fission.

When sepsis occurs, hepatocytes undergo pathological hypoxia, leading to disorder of mitochondrial oxidative phosphorylation, mitochondrial dysfunction and the production of massive ROS [29, 30]. And ROS is a key regulatory signal for the activation of NLRP3 inflammasome [31]. NLRP3 inflammasome consists of NLRP3, Apoptosis-associated speck-like protein containing a CARD and caspase-1 [31]. When NLRP3 is activated by ROS, ASC and caspase-1 are recruited to promote self-oligomerization to achieve close spatial distance of Pro-caspase-1, thereby forming mature caspase-1 through self-cleavage [31]. The precursor form of IL-β is cut by mature caspase-1, followed by maturation and secretion outside the cell. IL-β is involved in inflammatory response and promotes the release of massive downstream inflammatory factors [32]. Together with previous findings, our results indicate that GPR43 suppressed NLRP3 inflammasome through the inhibition of ROS production-induced mitochondrial fission in macrophage of sepsis. Collectively, GPR43 reduced mitochondrial damage to suppress NLRP3 inflammasome activity by the activation of ROS production in sepsis model. This experiment analyzed the GPR43 regulated NLRP3 inflammation and associated inflammation, however, we will further research GPR43 affect apoptosis and pyrocytosis in sepsis by the regulation of NLRP3 inflammasome.

The synthesis of massive ROS has been confirmed to promote the oxidative stress of podocytes, and apoptosis [33]. The massive synthesis of ROS induced by LPS must be mediated by the oxidation of p47phox by NADPH [34]. The translocation of p47phox from the cytoplasm to the cell membrane surface is an important process of oxidative stress [35, 36]. Here, we demonstrated that p47phox caused ROS production in the function of GPR43 in sepsis-induced inflammatory reactions model through the activation of NLRP3 inflammasome. Song et al. revealed that the inhibition of NLRP3 inflammasome activation depended on the reduction of p47phox by geniposide [37]. Thus, our results identify the p47phox as an important ROS sensing, anti-oxidant mechanism of GPR43 in phagocytes during of sepsis-induced NLRP3 inflammasome.

Recent studies have reported that PPARγ is expressed in various immune cells (airway epithelial cells, lymphocytes, etc.) [38]. PPARγ plays a role in inflammatory reaction, immune reaction, cell differentiation, cell proliferation and apoptosis [39]. PPARγ can regulate the transcription of target genes, inhibit the activation of immune cells and the expression of inflammatory factors, thus alleviating airway inflammation [40]. Recently, a large number of studies have shown that PPARγ has important antioxidant protection [41]. In addition, PPARγ activation can directly inhibit the production of ROS and promote the expression of antioxidant genes such as GST and SOD, thus antagonizing antioxidant stress [42]. Recent research reported that PAK4 interacted with PPARγ to regulate Nox1 in glioma [43]. Notably, this study showed that GPR43 induced PAK4 and PPARγ protein expressions and suppressed Nox1 protein expression in macrophage by LPS+ATP or mice model of sepsis. Ye et al. reported that Butyrate induced GPR43 expression to activate steroidogenesis through PPARγ in ovarian granulosa cells [44]. Thus, these results suggest that GPR43 is involved in the inactivation of NLRP3 inflammasome in sepsis model by the inhibition of ROS-induced mitochondrial damage via the induction of PPARγ function.

The NADPH oxidase of NOX family is an important source of cellular reactive oxygen species (ROS) [45]. NOX-derived ROS is involved in the hormone formation, chemical modification of matrix modification molecules, host defense, redox signaling, etc [46]. The activation of NOX-1 requires the translocation of the cytoplasmic subunits p40phox, p47phox, p67phox and Rac to the membrane subunit heterodimer cytochrome consisting of gp91phox and p22phox [47]. Molecular oxygen is transformed into superoxide anions, subsequently transforming into downstream metabolites with antibacterial activity, including hydrogen peroxide and hydroxyl anions [48, 49]. Myeloperoxidase (MPO) can convert hydrogen peroxide into low hydrochloric acid in neutrophils, which is a metabolite that effectively eliminates microorganisms [35, 50]. In this study, EBP50/Nox1/p47phox is involved in the activation of NLRP3 Inflammasome in sepsis model by the inhibition of GPR43 via PPARγ. Imad Al Ghouleh et al. identified that the binding of EBP50 to Nox-1 organizing subunit p47phox promoted smooth muscle ROS [47]. In line with these findings, we conclude that GPR43 weakened ROS production to suppresse NLRP3 inflammasome through the alleviation of mitochondrial damage in macrophage of sepsis by PPARγ/ Nox1/ EBP50/p47phox signaling. Such research will be also important for intensive understanding of how GRP43 plays an important role in inflammatory reactions of sepsis or other inflammatory diseases. This paper mainly researched sepsis-induced lung injury, it might regulate other tissue, and we will further research the effects of GPR43 in other visceral organ.

**Figure 9:**
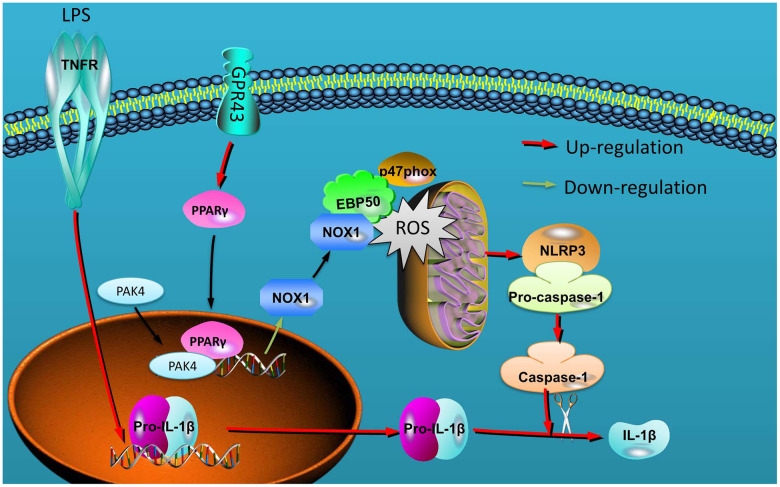
GPR43 is involved in the activation of NLRP3 inflammasome in sepsis model by ROS-induced mitochondrial damage via PPARγ.

Together with previous findings, our results indicate that GRP43 alleviated mitochondrial damage in macrophage by the promotion of ROS production to alleviate NLRP3 inflammasome of sepsis model by PPARγ/ Nox1/ EBP50/p47phox signaling (Figure 9). These findings may shed lights on understanding how GRP43 in alleviating the pathological processes of inflammatory reactions, and indicate that GRP43 have evolved a precise machinery in quantitative sensing of inflammatory reactions in many diseases. Moreover, the definition of GRP43 as mitochondrial damage sensed NLRP3 inflammasome may provide insights to the development of therapeutics for sepsis or inflammatory diseases such as rheumatism, carditis and so on.

## MATERIALS AND METHODS

### Animal experiment

Male C57BL/6 mice, WT mice or GPR43^-/-^ mice (5-6 weeks, 18-20 g) were housed separately under controlled temperature (22 ± 3° C), 50 ± 20% humidity, light-dark cycle of 12 h and free access to food and water. All animal experiments were performed in accordance with approved protocols for the BCM Institutional Animal Care and Usage Committee of Yijishan Hospital of Wannan Medical College. All C57BL/6 mice were obtained from Animal testing center of Qinglongshan (Nanjing, Suzhou, China). WT miceand GPR43^-/-^ mice were obtained from Model Animal Research Center of Nanjing University.

### Animal studies and cecal ligation and puncture (CLP) sepsis model

Mice were induced into sepsis using CLP model (n = 10). Mice of control group were alone raised (n = 10).

Mice of GPR43 agonist group (n = 10) were treated with 4-CMTB (10 mg/kg, i.p., MedChemExpress, China) for 24 h after induction of CLP model.

Mice of control group (n = 10) were treated with normal saline (10 mg/kg, i.p.) for 24 h after induction of CLP model.

All mice were anesthetized by intraperitoneal injection of 50 mg/kg pentobarbital sodium (i.p., MedChemExpress, China) and subjected to laparotomy followed by extracorporeal cecum mobilization and ligation. Mice were sacrificed and then lung tissue samples were immediately weighed (W). Lung tissue samples were dried at 70° C for 48 h to constant weight (D). Interstitial fluid of lung was evaluated using the calculated W/D ratio.

### Hematoxylin-eosin (H&E) staining

After mice sacrificed, lung tissue was collected and fixed with 4% paraformaldehyde for 24 h at room temperature. Lung tissue samples fixed with paraformaldehyde were paraffin-embedded. Samples were cut into 5 *μ*m sections using a paraffin slicing machine and stained with hematoxylin and eosin. Light microscopy (BH3-MJL; Olympus Corporation, Tokyo, Japan) observed lung tissues.

### Enzyme-linked immunosorbent assay (ELISA)

After vitro experiment or vivo experiment, TNF-α (H052), IL-1β (H002), IL-6 (H007), IL-10 (H009), IL-12 (H010) and IFN-γ (H025) protein levels in serum, lung tissue and cultured cell were detected using an ELISA kit according to manufacturer’s instructions (Nanjing Jiancheng Bioengineering Research Institute). Absorbance was obtained with a microplate reader (ELx800; General Electric) at 450 nm. ROS level was detected using an ELISA kit (S0033S) according to manufacturer’s instructions (Beyotime). Absorbance was obtained with a microplate reader (ELx800; General Electric) at 405 nm.

### RT-PCR

Cells were extracted for total RNA using RNAiso Plus reagent (Takara, Japan). cDNA was synthesized from 2.5 μg total RNA using Superscript II reverse transcriptase (Applied Biosystems, CA, USA). The mRNA expression levels were quantified by quantitative real-time PCR with SYBR green PCR mastermix (Takara, Japan). The following primers were used: GPR43: 5′-ACAGTGGAGGGGACCAAGAT-3′, 5′-GGGGACTCTCTACTCGGTGA-3′; β-Actin, 5′-TATGCCAACACAGTGCTGTCTGG-3′ and 5′-TACTCCTGCTTGCTGATCCACAT-3′. The PCR cycling conditions were 50° C for 15 s and 60° C for 60 s. The mRNA expression levels were quantified by analyzed against the endogenous genes of β-actin as an internal control.

### Western blot

Cells and tissue samples were lysed for protein extraction using RIPA assay. The concentration of each protein sample was determined by a BCA (bicinchoninic acid) kit. Equal amounts (50 mg) of protein were separated by 10% sodium dodecyl sulfate-polyacrylamide gel electrophoresis and transferred to a polyvinylidene fluoride membrane (Bio-Rad, Hercules, CA, United States). Membranes were blocked using 5% non-fat dry milk with TBST buffer for 1 h, then incubated overnight at 4° C with GPR43 (1:1000, ab131003, Abcam), MFN2 (1:500, ab205236, Abcam), PPARγ (1:1000, ab178860, Abcam), NOX-1 (1:1000, ab121009, Abcam), EBP50 (1:1000, ab3452, Abcam), p47phox (1:1000, ab166930, Abcam), NLRP3 (1:1000, 15101, Cell Signaling Technology, Danvers, MA, US), Caspase-1 (1:1000, sc-392736, Santa Cruz Biotechnology), IL-1β (1:1000, 12242, Cell Signaling Technology, Danvers, MA, US), and β-Actin (1:5000, sc-47778, Santa Cruz Biotechnology). After washing, membranes were probed further with horseradish peroxidase-conjugated goat anti-rabbit or anti-mouse IgG (1:5000, Santa Cruz Biotechnology). After washing with TBST for 15 min, immunoreactive bands were exposed by enhanced chemiluminescence method (Thermo Fisher Scientific, Waltham, MA, USA).

### Cell transfection and *in vitro* model

RAW264.7 cell (murine macrophage cell) was purchased from Shanghai cell bank of Chinese Academy of Sciences (Shanghai, China) and maintained in DMEM (Gibco) supplemented with 10% FBS (Gibco) under a humidified 5% (v/v) CO2 atmosphere at 37° C. The transfections were performed using Lipofectamine 2000 (Thermo Fisher Scientific). The GPR43 plasmids (0.4 μg/ml, 5′-CATGGCTTACATCATCATCT-3′ and 5′-CTACAGAGTAGCAGTTTCCC-3′) or the si-GPR43 (sc-77339, Santa Cruz Biotechnology), si-NLRP3 (sc-45469, Santa Cruz Biotechnology), si-p47phox (sc-29422, Santa Cruz Biotechnology) and NOX-1 siRNAs (20 nmol/ml, sc-43939, Santa Cruz Biotechnology) were transfected in the serum-free and antibiotic free media. After 48 h of transfection, cells were inducted with 100 ng/mL of LPS (MedChemExpress, China) for 4 h and 2 mM of ATP (MedChemExpress, China) for 30 min as document [51]. Next, after 48 h of transfection, cells were inducted with 4-CMTB (10, 20 and 40 μM) and 100 ng/mL of LPS for 4 h and 2 mM of ATP for 30 min.

### Cell culture and treatment

Scalp needle (No. 7) was inserted into the trachea from centripetal direction and fixed with silk thread. Before residual lung gas pump back, RPMI 1640 (Gibco) supplemented with 5 % FBS (Gibco) slowly pushed into the trachea by syringe and lung tissue was put in sterile petri dish. 5 ml of RPMI 1640 supplemented with 5 % FBS was added into lung tissue, and lung tissue was grinded using centrifugal tube to prepare single cell suspension. Single cell suspension was added into centrifugal tube using mesh and discard the supernatant at 1500 r/min for 15 min. RPMI 1640 supplemented with 20 % FBS was added into cell and bilayer lymphocyte separation fluid was added into cell. After centrifuging at 2000 r/min for 20 min, cells of the upper and lower cell separation fluid interfaces were collected and fixed with Electron microscope fixative at room temperature for 30 min at darkness. Then, cell was observed using a Hitachi H7650 transmission electron microscope (Tokyo, Japan).

Cristae density was calculated by using Image pro for area void of cristae as previously described [52]. Mitochondrial size was measured using tracing individual mitochondrion after calibration for distance (minimum of 40 mitochondria).

### Mitochondrial membrane potential assay and mitochondrial permeability transition pore assay

1x 10^5^/well of RAW264.7 cell was seeded into a 96-well plate and 100 μL JC-1 probe solution (C2006, Beyotime Biotechnology) was added into every well as previously described [52]. Absorbance was measured using a fluorescent reader (Synergy H1 Microplate Reader, Bio Tek, Winooski, USA).

1x 10^5^/well of RAW264.7 cell was seeded into a 96-well plate, diluted calcein-AM solution to 500 nM with solution buffer, incubated cells with for 20 min at 37° C, and added with 50 μL CoCl2 solution and incubated for 10 min. Absorbance was measured using the plate under Ex490/Em515 nm with fluorescent reader (Synergy H1 Microplate Reader, Bio Tek, Winooski, USA) as previously described [52].

### Immunoprecipitation (IP)

In IP, the lysate protein (500 μg) was mixed with 2 μg protein G agarose antibody (16e266, Millipore, Billerica, MA, USA), and incubated and rotated overnight at 4° C. Proteins were collected as IP production after washing buffer three times. The succinylation signal was detected and separated on a 10% sodium dodecyl sulfate-polyacrylamide gel electrophoresis (SDS-PAGE) and subsequently transferred onto a polyvinylidene difluoride (PVDF) membranes (Millipore, USA), and incubated with primary antibodies: NOX-1 (1:100, ab121009, Abcam), EBP50 (1:100, 3394, Abcam or 1:50, sc-271552, Santa Cruz Biotechnology), p47phox (1:100, ab166930, Abcam or 1:50, sc-17845, Santa Cruz Biotechnology) overnight at 4° C. Membranes were washed with TBST and incubated with HRP-conjugated secondary antibody (sc-2004, sc-2005, 1:2000, Santa Cruz Biotechnology) at room temperature for 1 h. Membrane was developed with ECL (Promega, USA).

### Immunofluorescence

Cell were washed with PBS and fixed with 4% paraformaldehyde supplemented with 0.25% Tris-X100 at room temperature for 10 min. After blocking with PBS supplemented with 5% BSA for 2 h at room temperature, cells were incubated with NOX-1 (1:200, ab55831, Abcam), EBP50 (1:1000, 3394, Abcam or 1:200, sc-271552, Santa Cruz Biotechnology), p47phox (1:200, ab166930, Abcam or 1:200, sc-17845, Santa Cruz Biotechnology) at 4° C overnight. Cells were incubated with secondary peroxidase conjugated goat anti-rabbit IgG (1:100, Santa Cruz Biotechnology) antibody for 2 h at room temperature, after washing with PBST for 15 min. Cells were stained with DAPI for 15 min at darkness, after washing with PBST for 15 min. Cell samples were observed using fluorescence microscope (Zeiss Axio Observer A1, Germany).

### Statistical analysis

Statistical analyses were performed using GraphPad Prism 5 software. The quantitative data were represented as mean ± standard deviation (x̄ ±s). *p*<0.05 was considered statistically significant. Differences between two groups were tested by the Student’s t-test. ANOVA followed by Dunnett’s test was used to compare three and more groups.

### Data availability

If the data are all contained within the manuscript and/or Supporting Information files, enter the following: All relevant data are within the manuscript and its Supporting Information files.

### Credit authorship contribution statement

Zhichen Pu: Conceptualization, Methodology, Investigation, Formal analysis, Writing-original draft, Visualization, Project administration. Wusan Wang: Conceptualization, Writing - original draft, Visualization. Maodi Xu: Methodology, Visualization. Haitang Xie: Writing - review &editing, Supervision, Funding acquisition. Weiwei Zhang: Writing - review and editing, Supervision, Funding acquisition.

## Supplementary Material

Supplementary Figures
