# Supplementary material for: GPR43 regulation of mitochondrial damage to alleviate inflammatory reaction in sepsis
Source: Aging (Albany NY). 2021 Sep 28;13(18):22588–610. doi: 10.18632/aging.203572 (PMC8507289; doi:10.18632/aging.203572)
Supplement: Supplementary Figures [file aging-13-203572-s001.pdf]

## SUPPLEMENTARY FIGURES

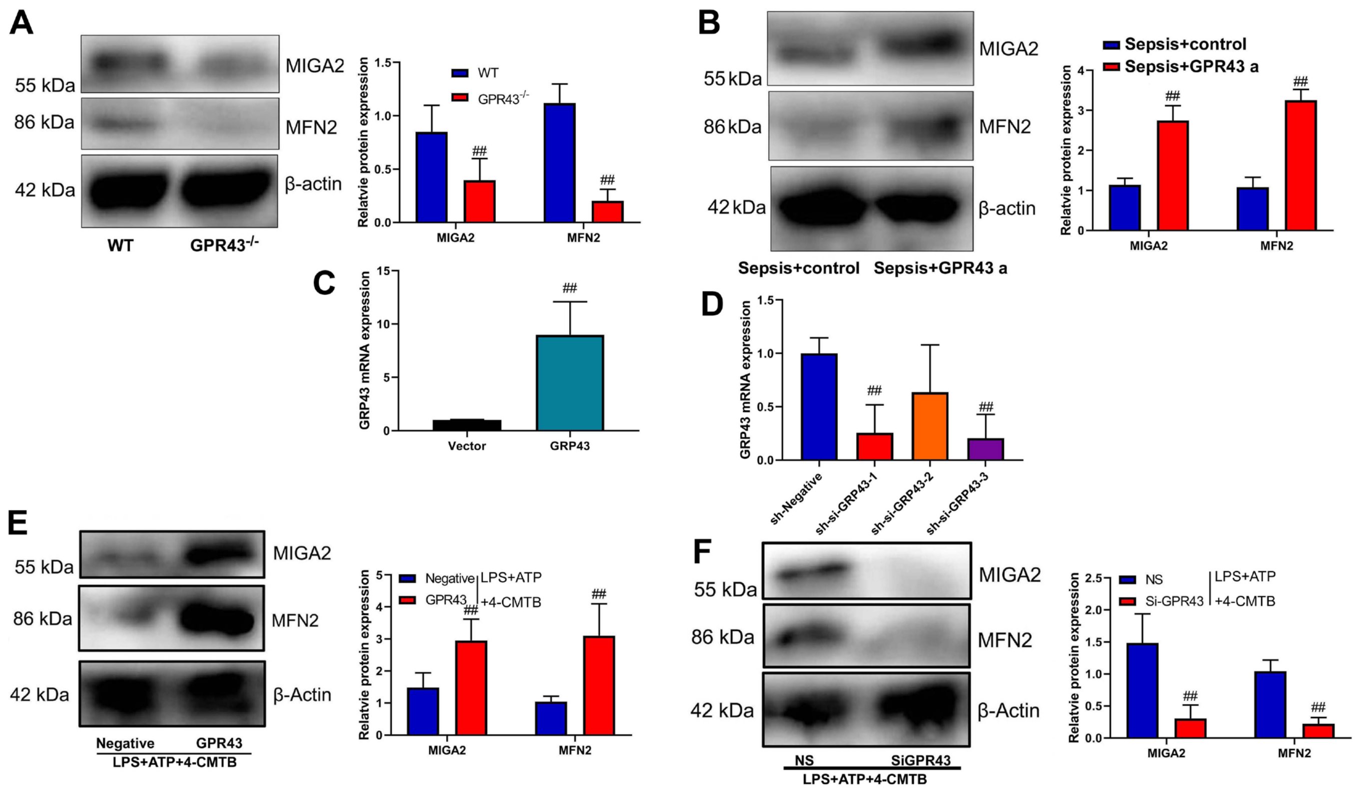


**Supplementary Figure 1. Mitochondrial fission and GPR43 expression *in vitro* model.** MIGA2 and MFN2 protein expressions in GPR43$^{-/-}$ mice with CLP (**A**) for 24 h; MIGA2 and MFN2 protein expressions in CLP mice with GPR43 agonist (**B**) for 24 h; GPR43 expression *in vitro* model by over-expression of GPR43 (**C**) for 24 h; GPR43 expression *in vitro* model by down-regulation of GPR43 (**D**) for 24 h; MIGA2 and MFN2 protein expressions in macrophage by up-regulation of GPR43 and LPS+ATP+GPR43 agonist (**E**) for 24 h; MIGA2 and MFN2 protein expressions in macrophage by down-regulation of GPR43 and LPS+ATP+GPR43 agonis (**F**) for 24 h; Sepsis+control, CLP mice with normal saline; Sepsis+GRP43 a, CLP mice with i GPR43 agonist (4-CMTB, 10 mg/kg, i.p.); WT, WT mice with CLP; GPR43$^{-/-}$, GPR43$^{-/-}$ mice with CLP; Negative, negative control; Negative, negative control; GPR43, over-expression of GPR43; NS, si-negative control; Si-GPR43, down-regulation of GPR43; LPS+ATP+4-CMTB, macrophage by treated with LPS+ATP+4-CMTB. ##p<0.01 compared with WT mice with CLP or ##p<0.01 compared with CLP mice with normal saline; ##p<0.01 compared with negative control or si-negative control.

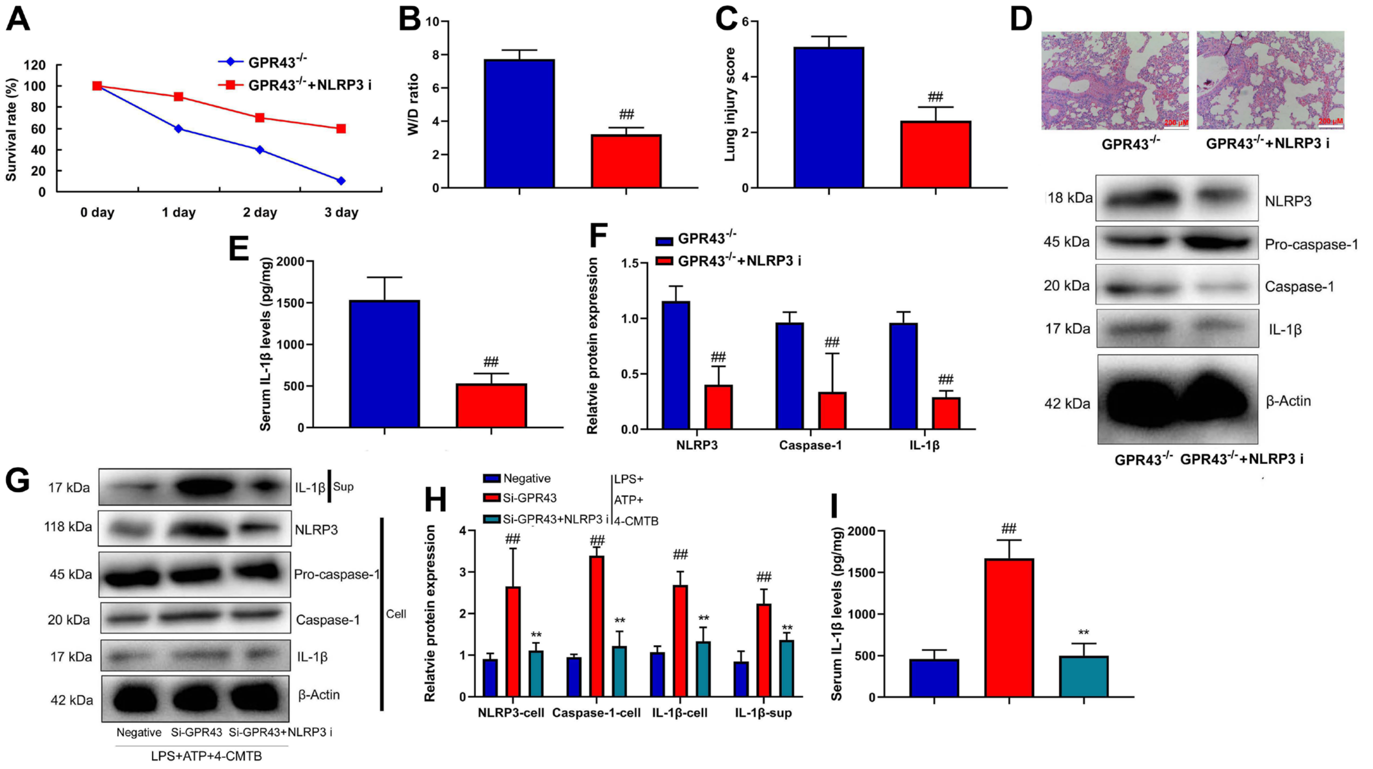


**Supplementary Figure 2. GPR43 is involved in the activation of NLRP3 Inflammasome in sepsis model.** Survival rate (**A**) in GPR43$^{-/-}$ mice of CLP with NLRP3 inhibitor for 72 h; W/D rate (**B**), lung injury score (**C**), lung tissue using HE staining (**D**), serum IL-1β levels (**E**), EBP50/GPR43/NLRP3/caspase-1/IL-1β protein expressions (**F**) in GPR43$^{-/-}$ mice of CLP with NLRP3 inhibitor for 24 h; NOX-1/EBP50, GPR43, NLRP3, Caspase-1 and IL-1β protein expressions in cells and IL-1β protein expression in supernatant (**G**, **I**), IL-1β levels (**H**) in macrophage by down-regulation of GPR43 and LPS+ATP+GPR43 agonist for 24 h. GPR43$^{-/-}$, GPR43$^{-/-}$ mice with CLP; GPR43$^{-/-}$+ NLRP3 i, GPR43$^{-/-}$ mice of CLP with NLRP3 inhibitor; Negative, negative control; Si-GPR43, down-regulation of GPR43; NLRP3 i, NLRP3 inhibitor; LPS+ATP+4-CMTB, macrophage by treated with LPS+ATP+4-CMTB. ##p<0.01 compared with GPR43$^{-/-}$ mice with CLP or GPR43$^{-/-}$ mice with CLP; **p<0.01 compared with down-regulation of GPR43.

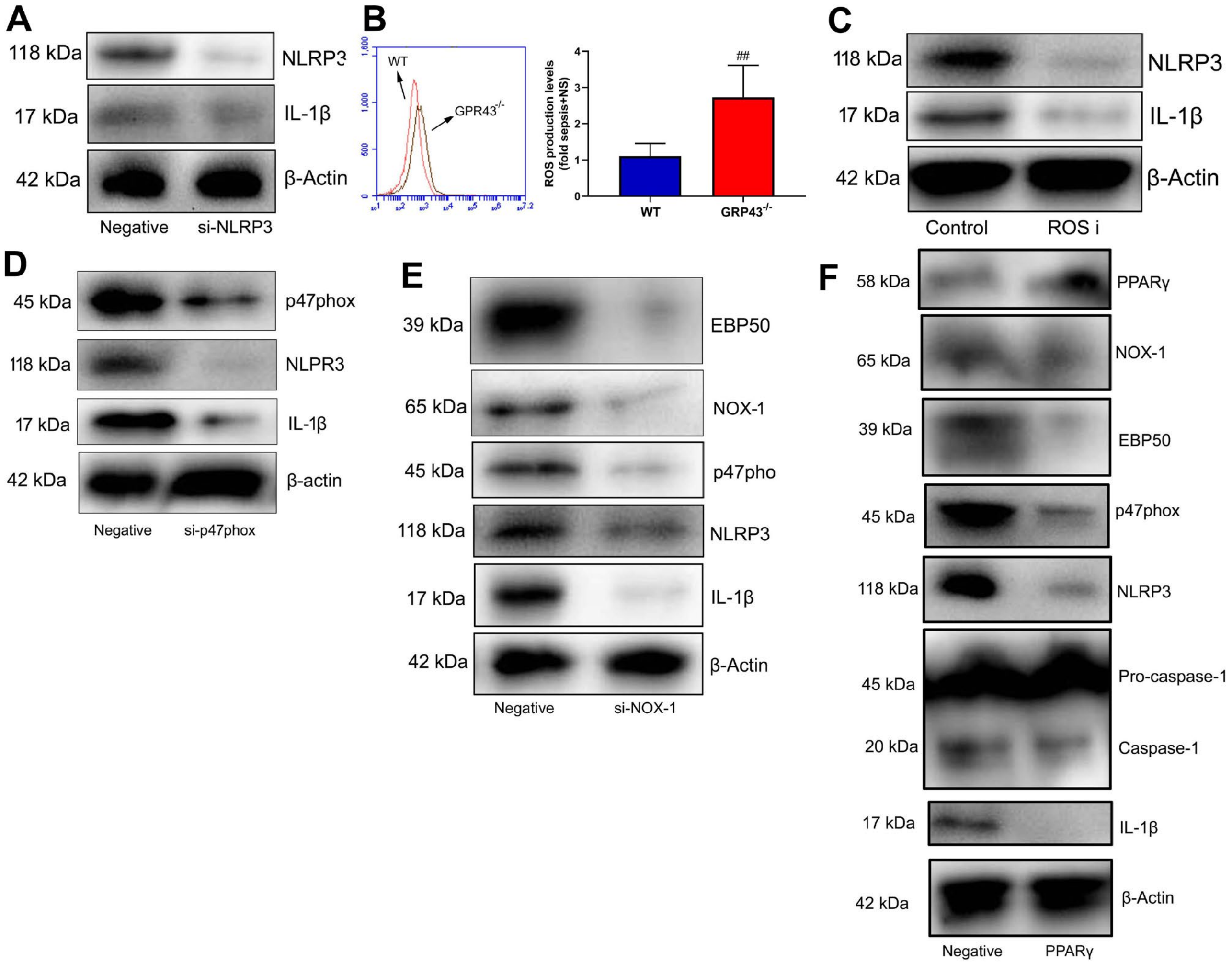


**Supplementary Figure 3. Expression of protein *in vitro* model and ROS production.** NLRP3/ IL-1β protein expressions *in vitro* model by si-NLRP3 (**A**); ROS production levels in macrophage of CLP mice with OM (**B**) for 24 h; NLRP3/ IL-1β protein expressions *in vitro* model by ROS inhibitor (**C**); p47phox/NLRP3/ IL-1β protein expressions *in vitro* model by si-p47phox (**D**); EBP50/NOX-1/ p47phox/NLRP3/ IL-1β protein expressions *in vitro* model by si-p47phox (**E**); PPARγ/EBP50/NOX-1/ p47phox/NLRP3/ IL-1β protein expressions *in vitro* model by PPARγ a (**F**). ##p<0.01 compared with negative or control or DMSO group.

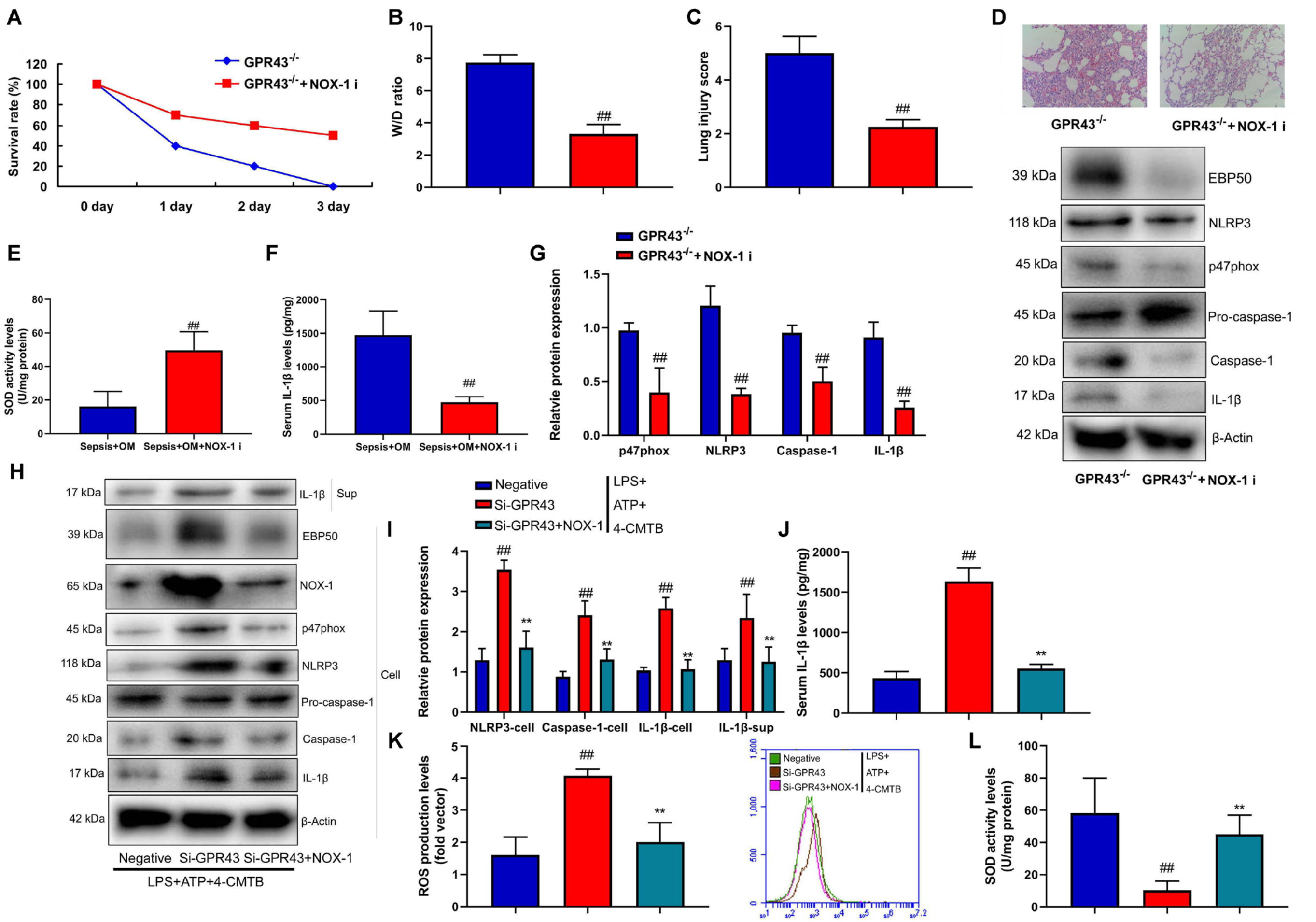

**Supplementary Figure 4. GPR43 is involved in the activation of NOX-1 in sepsis model.** Survival rate (**A**) in CLP mice with OM or NOX-1 inhibitor for 72 h; W/D rate (**B**), lung injury score (**C**), lung tissue using HE staining (**D**), SOD activity levels (**E**), serum IL-1β levels (**F**), EBP50/GPR43/NLRP3/caspase-1/IL-1β protein expressions (**G**) in CLP mice with OM or NOX-1 inhibitor for 24 h; NOX-1/EBP50, p47phox, NLRP3, Caspase-1 and IL-1β protein expressions in cells and IL-1β protein expression in supernatant (**H**, **I**), IL-1β levels (**J**), ROS production level (**K**), and SOD activity levels (**L**) in macrophage by down-regulation of GPR43 and LPS+ATP+GPR43 agonist for 24 h. GPR43$^{-/-}$, GPR43$^{-/-}$ mice with CLP; GPR43$^{-/-}$+ NOX-1 i, GPR43$^{-/-}$ mice of CLP with NOX-1 inhibitor; Negative, negative control; Si-GPR43, down-regulation of GPR43; NOX-1 i, NOX-1 inhibitor; LPS+ATP+4-CMTB, macrophage by treated with LPS+ATP+4-CMTB. ##p<0.01 compared with GPR43$^{-/-}$ mice with CLP or GPR43$^{-/-}$ mice with CLP; **p<0.01 compared with down-regulation of GPR43.
